# Pulse Invariability in Menstruation, Regardless of Posture

**Published:** 1887-05

**Authors:** 


**Pulse Invariability in Menstruation, Regardless of Posture.**—The fact that the pulse of the normal male beats from ten to fifteen strokes more per minute when the body is in a vertical position than when lying down, has long been recognized, and until a very recent period it was assumed that the same difference existed in the pulse of the female. Graves first pointed out that in cases of cardiac hypertrophy the pulse remains constant in all positions. More recently, Jorissenne discovered that in pregnancy the same constancy exists in the female, and suggested this fact as a diagnostic test of that condition. *La France Medicale* now announces that M. P. Louge, intern of the Marseilles Hospital, has discovered that in women there exists during the menstrual flow the same constancy of pulse in all positions of the body. It is exceedingly difficult to account for this phenomenon by any known physiological law. Cardiac hypertrophy cannot be invoked, and the only hypothesis that there is an augmentation of the tension of the blood during menstruation—a suggestion which seems to be supported by certain clinical phenomena of the catamenial period.—*St. Louis Médical and Surgical Journal.*

